# When innovation meets patient blood management – a new way to see bleeding

**DOI:** 10.1016/j.htct.2024.07.002

**Published:** 2024-09-07

**Authors:** Guilherme Rabello, Rosangela Monteiro, Bianca Meneghini, Fabio Biscegli Jatene

**Affiliations:** a InovaInCor Innovation Department, Heart Institute, School of Medicine, University of Sao Paulo (USP), Sao Paulo, SP, Brazil; b Department of Cardiovascular Surgery, Heart Institute, School of Medicine, University of Sao Paulo (USP), Sao Paulo, SP, Brazil

**Keywords:** Patient blood management, Bleeding, Hemostasis, Blood coagulation, Validated intraoperative bleeding (VIBe) scale

## Abstract

The first step in innovation is to identify a problem of real relevance and systematically address it to deliver a sophisticated and viable solution. Disruptive innovation is a process where technology, products, or services are transformed or replaced by a better innovative solution. This superiority must be perceived by users as being more accessible, simple, or convenient. Patient Blood Management (PBM) suggests the notion of the timely application of evidence-based medical and surgical concepts designed to maintain hemoglobin concentration, optimize hemostasis and minimize blood loss thus improving patient outcomes, that is, they are aimed at changing patient care, assisting healthcare professionals in disease treatment and cure as well as risk reduction. Thus, innovation in PBM is a new frontier to be pursued. The management of patient's blood and preparation for surgical procedures is an enormous challenge that helps minimize anemia and control blood loss during hospitalization, ensuring they are discharged in adequate clinical conditions. Until 2016, there was no standard definition or classification for the severity of intraoperative bleeding or hemostasis. The development of a PBM program when combined to the development of a bleeding scale such as the validated Intraoperative Bleeding (VIBe) Scale, represents a new solution that balances perioperative blood loss and more importantly, enables a critical cultural change which can be useful to help surgeons communicate anticipated hemostatic needs throughout a case and therefore enhance efficiency leading to better outcomes.

## Innovation

Innovation is the creation of a new viable offer, be it a product, process or service. Innovations may be incremental, radical or disruptive depending on the impact that new processes, products and business models have in the market.^1^

The innovation process requires identifying the problem that really matters and systematically overcoming it to offer an elegant and viable solution. Disruptive innovation is a process whereby technology, products or services are modified or replaced by a better innovative solution. This superiority needs to be perceived by users as being more accessible, simple or convenient.

While the invention of new technologies, practices, and care models are relevant moments in healthcare, technical innovation is only half of the story. Replicating and scaling a healthcare innovation that worked successfully in one study or environment and then making it work in a new context is not simple, much less automatic.

Perhaps many of us believe that innovation demands complexity, something too revolutionary to be understood, the field of brilliant and incomprehensible minds, however, simplicity is essential for an innovation to achieve its intended success, even though it is not synonymous to simple ideas. It has to do with taking complex ideas and concepts and being capable of turning them into a simple solution. It is about reducing the complexity to something easy to understand and use in practice. But simplicity, just as successful innovations, is a challenging target.

## Patient blood management (PBM) as an innovative process

Each year, over 60,000 Americans die from bleeding causes.^2^ Worldwide, that number is nearly two million, mainly due to trauma cases. A reasonable portion of patients who undergo surgical procedures formerly have active bleeding conditions or experience iatrogenic bleeding caused by the surgical intervention. Accurate recognition and prompt actions using the best possible technique are essential for patient safety and survival, the foundation of PBM.

The denomination of PBM emerged in 2005 and suggested the notion of the timely application of evidence-based medical and surgical concepts designed to maintain hemoglobin concentration, optimize hemostasis, and minimize blood loss in an effort to improve patient outcomes. ^3^ In 2021, the Society for the Advancement of Patient Blood Management (SABM) applied this concept focusing on reducing the use of blood components through a multidisciplinary and multimodal strategy to improve patient outcomes.^4^

In 2010, the World Health Organization (WHO) endorsed PBM^5^, and the fourth strategic objective of the '’WHO Framework of Action for Blood Products 2020–2023′' launched in February 2020, requires the effective implementation of PBM. ^6^ In October 2021, the WHO published a Policy Brief urging all member states to act quickly through their ministries of health to adopt a national PBM policy, install the necessary governance, and reallocate resources to improve the health status of the population and individual patient outcomes, reducing overall health expenditures.^7^

**Figure 1:**
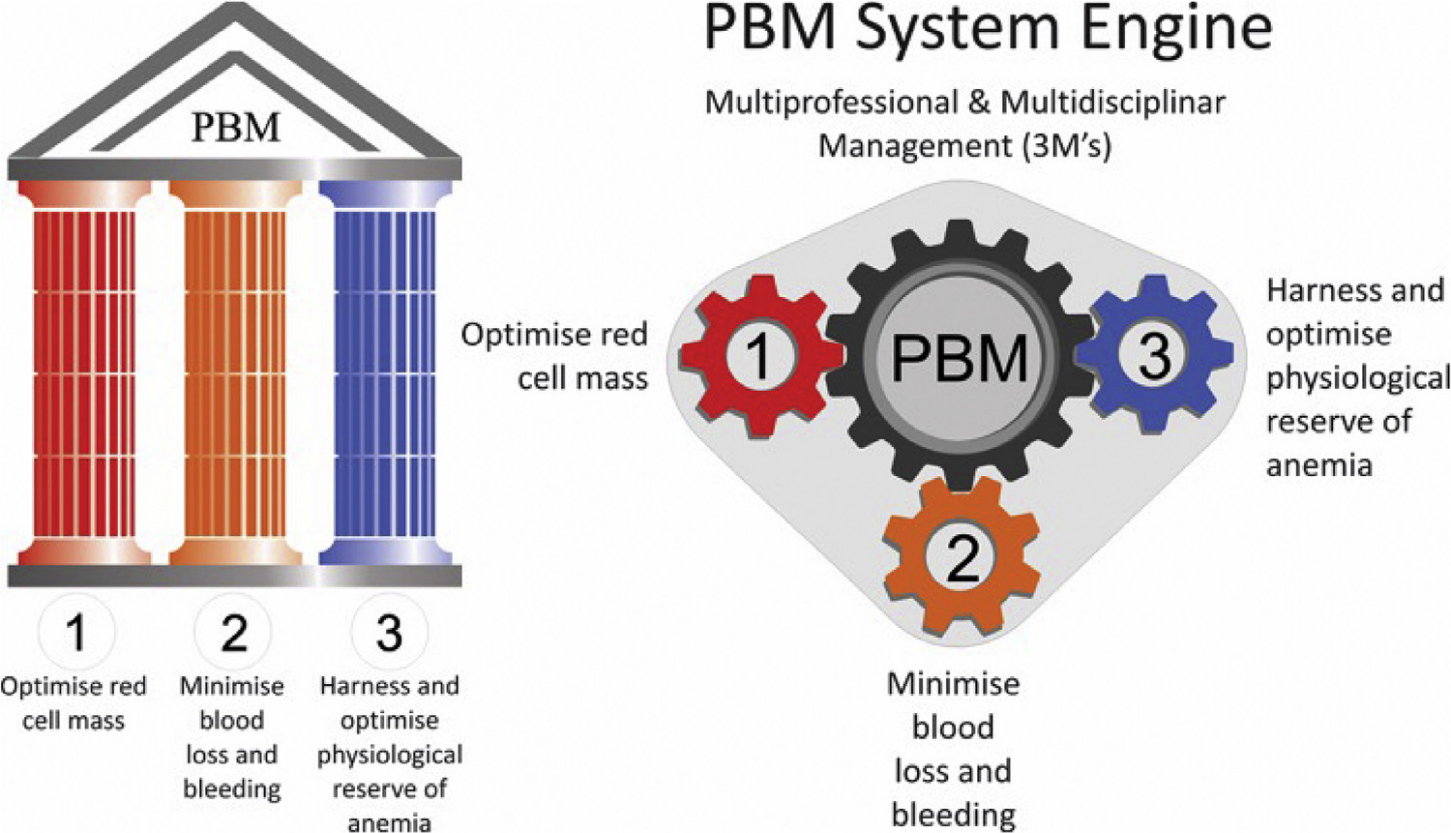
The 3 pillars of PBM with its System Engine (3M's) (Adapted from Hoffman et al.^8^).

Strategies comparable to the development of the PBM program are propagating worldwide to add a systemic view of coordinated care while promoting the synergistic action of clinical and surgical techniques and methods shown in Figure 1.^8^

Thus, innovation in PBM is a new frontier to be pursued. Advances in blood conservation, pharmacologic manipulation, engineered blood products and recombinant growth factors represent a safer and more effective alternative to blood transfusions. However, there is an enormous challenge in patients' blood management and preparation for surgical procedures, minimizing anemia and controlling blood loss during hospitalization to ensure patients are discharged in adequate clinical conditions.

## The VIBe Scale

Clinical studies until 2016 showed no standard definition or classification for the severity of intraoperative bleeding or hemostasis, partially due to the lack of consensus to define severity of bleeding and partly due to the lack of threshold values.

Thus, researchers have proposed the development of a validated bleeding severity scale using standardized criteria that would ensure that patients are not subject to unnecessary risk (e.g., failure, delay, or improper use of treatments for hemostasis). With standardized criteria, outcomes and clinical trial results become comparable and help us to establish the relative effectiveness of the respective hemostatic strategies.^9^ The VIBe Scale classification is based on visual appearance in the surgical field and is correlated with the blood loss rate and potential tissue damage.^9^

It meets all Food and Drug Administration (FDA) criteria for a clinician-reported scale: the ability to detect change, clarity, construct validity, relevance, repeatability, reproducibility, response range, and usability.^9^

**Figure 2:**
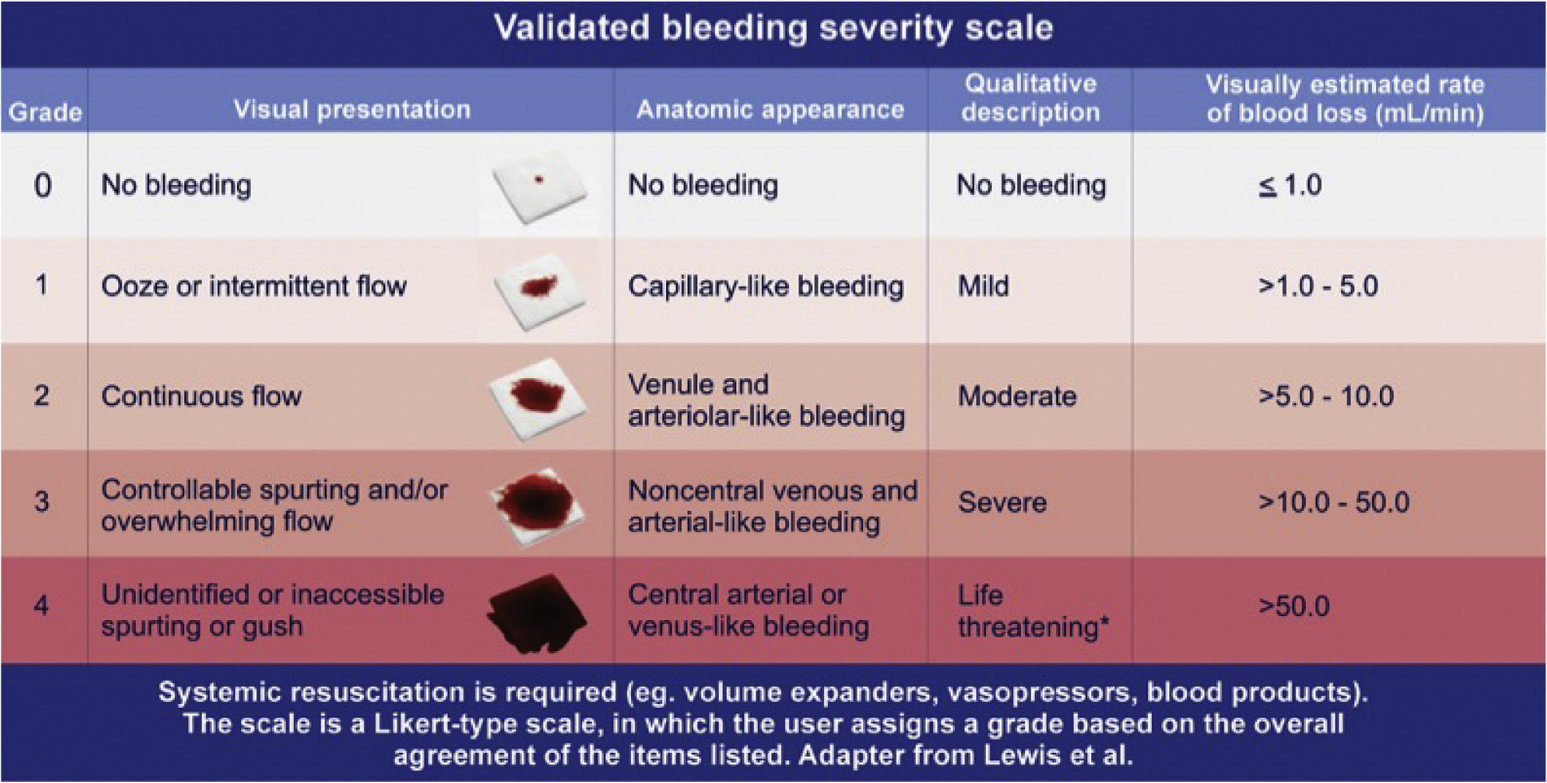
VIBe Scale Rating. Copyright 2023 Baxter Healthcare Corporation, used by permission.

Figure 2 shows the classification levels of the VIBe Scale (0 to 4).^10^ Since it is a simple, visual, and objective scale, its adoption is more feasible if we compare it with other bleeding assessment techniques and systems that depend on equipment, inputs, and the specialized training of professionals. This is in general, a determining factor in the scalability and replicability of the proposed innovation, be it a product or a process.

**Figure 3:**
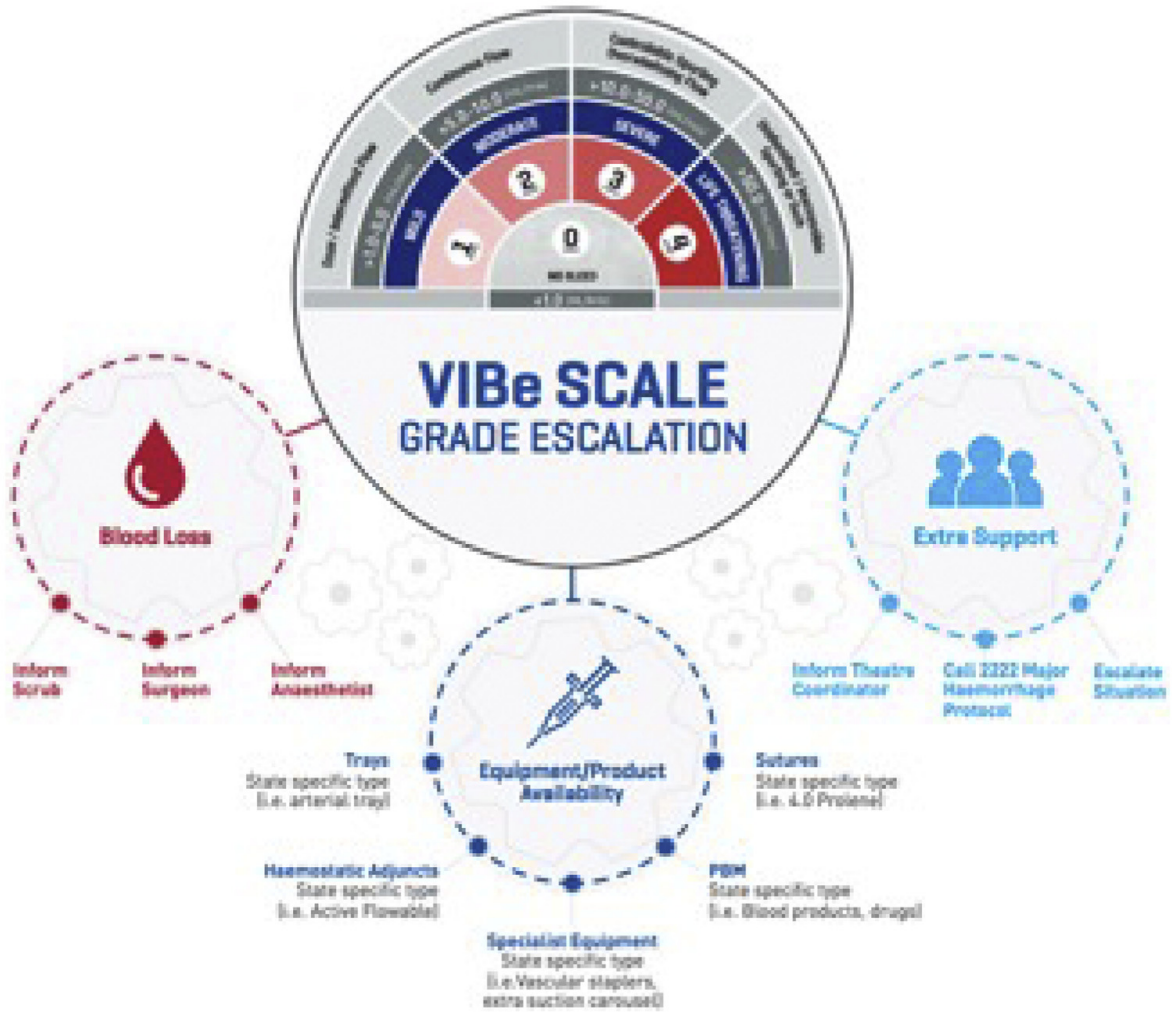
VIBe Scale grade escalation. Copyright 2023 Baxter Healthcare Corporation, used by permission.

The applications of the VIBe Scale for rapid identification of the degree of bleeding as alternatives to the use of equipment, products and approaches to hemostasis as well as the support from other professional teams in patient care is shown in Figure 3.

A few studies have been conducted aiming to validate the scale in different fields. A multicenter study evaluated the applicability and reproducibility of the VIBe Scale in liver surgery and, through video observation, the authors concluded that the bleeding scale has high interobserver and intraobserver concordance, implying that the VIBe Scale is a useful tool in liver surgery.^11^

Spine surgeons have also applied the VIBe Scale and scored videos depicting surgical bleeding. The researchers highlighted the reliability of the scale and potential to quantify intraoperative blood loss in spine surgery. They also reported that the preoperative use of the scale may support strategies to control bleeding and hemostasis in complex surgical procedures.^12^

A most recent study was published performing a human factor verification on the bleeding severity scale for use in the clinical practice across a considerably larger international group of surgeons of different specialties, as general surgery, cardiac surgery, neurosurgery, gynecology, urology and spine surgery (both neurosurgical and orthopedic). As a result, the robust data demonstrates the VIBe Scale is applicable in the real world clinical practice. The development of a universal bleeding scale across multiple surgical specialties is both needed and feasible to provide better patient care.^13^

## Cultural change

Experienced physicians often find a level of comfort with how they conduct their day-to-day clinic.^14^ The brain can resist change, nonetheless, with the right attitude, physicians can implement the changes they need to improve their practice and better cope with the updates in medicine.

The delay in changing clinical protocols in hospitals, even after the publication of new guidelines, which have been reviewed and updated by medical societies, is a proven challenge. As reported by Rubin, it takes an average of 17 years for new medical recommendations to become part of the daily clinical practice.^15^

Making changes takes work and often involves spending money, diverting staff from their daily activities, changing rooted cultural or professional norms, and taking risks.

There is rarely a single right way to deal with a complex problem. Hence, conflicting perspectives should be seen as the basic ingredient for multifaceted solutions. This is why we need to understand how we should change the way we see surgical bleeding and at the same time, the alternatives for its clinical approach. It is more than a technical change; it entails a cultural modification and process innovation. The mindset of hemostasis and bleeding needs to change! As we already know, we have a huge number of patients regularly submitted to surgical procedures in suboptimal conditions, especially when it comes to preoperative anemia and coagulopathies.^16^

Additionally, surgical protocols that contemplate blood component transfusions (mainly red blood cells and platelets) as the standard intervention to correct iatrogenic anemia and intraoperative hemorrhage are still prevalent, despite the growing evidence of transfusion risks and their side effects. This only shows we have a long way to go before we can change the culture among surgeons and anesthesiologists where transfusions are still the 'fastest and safest' way to reverse anemia and coagulopathies_._^1^^,^^17^

The new definition of PMB launched in 2022 emphasizes a patient-centered, systematic, evidence-based approach to improving patient outcomes by managing and preserving the patient's own blood while promoting safety and the empowerment of patients. Thus, reflection on the traditional transfusion practice has been encouraged, and in particular on the emphasis of meticulous hemostasis (technical expertise) and strategic hemostasis approaches (logical expertise) through the use of active and passive hemostatic products (which include, for example, topical sealants, glues and antifibrinolytics).^4^ However, preventing patients from bleeding is not feasible, reasonable, or even possible. It is an inherent part of the surgical process, especially the iatrogenic damage of bleeding. Nevertheless, changing our approach to learn how to quickly identify and classify bleeding, based on a simple and effective severity scale, in a way that ensures patients are not facing unnecessary risk is our ethical and professional duty.

Accordingly to Shander, a leader in PBM, “old habits die hard”, focusing on change of medical culture supported by evidence-based medicine.^18^

Given this challenge, we understand the reason for the role of the VIBe Scale as a potential cultural change and innovation within the PBM movement.

The ‘wait and watch’ concept or the control of bleeding by the use of blood component transfusions is still the standard. It basically resorts to platelets for hemostasis and red blood cell transfusions to mitigate anemia. Despite being a routine and usual protocol in the majority of surgical centers worldwide, both approaches are increasingly questioned, given their risks and weak evidence of clinical effectiveness.^19^, ^20^, ^21^

In the context of the progressive adoption of PBM as the new norm of concerned care, when actively seeking to manage and preserve the patient's blood, the use of a simple, accessible, and effective scale to validate the degree of bleeding can increase the safety of the surgical process, speed up actions aimed at rapid and effective hemostasis, reduce risks and improve clinical outcomes.^22^

We do not need any technologically complex equipment, costly to operate and dependent on scarce resources, as we already have the most ingenious and sophisticated system available, human eyes and our trained cognitive intelligence, in addition to a simple gauze to measure bleeding. Therein lies the beauty of complexity with simplicity, the true ingenuity of the VIBe Scale that makes it a distinctive innovation in modern Medicine. Although implementation requires some commitment from surgeons after completing training, they can assess and report the severity of bleeding in a reproducible and accurate manner, not only in the setting of liver surgery, as shown previously, but also in other surgical specialties.^11^^,^^12^

In this sense, the VIBe Scale should be seen as a disruptive innovation that converges with the objective of PBM and patient-centered medicine, allowing any surgical team to use it for prompt evaluation of the degree of bleeding.

Renowned Professor Peter Drucker said: “for an innovation, to be effective, has to be simple and it has to be focused. It must do only one thing, otherwise it confuses. If it's not simple, it won't work. Everything new has problems; if complicated, it cannot be repaired or fixed. All effective innovations are incredibly simple. In fact, the highest praise an innovation can receive is when people say, ’That's obvious. Why didn't I think of that?'”.^23^

Using the innovation process to find a new solution that helps equate perioperative bleeding with PBM is something the VIBe Scale sets out to do and manages to deliver.

In conclusion, the use of healthcare innovation tools improves patient processes and outcomes. The development of a PBM program, when combined to the creation of a bleeding scale such as the VIBe Scale, represents a new solution that not only balances perioperative blood loss, but also enables an important cultural change, and can be useful to help surgeons communicate anticipated hemostatic needs throughout a case, enhancing efficiency and thus leading to better outcomes.

## Funding

The present study received funding from the Baxter Healthcare Corporation.

## Disclosures

Guilherme Rabello | Payments for lectures, manuscript preparation or development of educational materials from Baxter, Abbott, Vifor Pharma, Nestlé and Cristalia.

## Conflicts of interest

None.
